# Liraglutide improves the beta-cell function without increasing insulin secretion during a mixed meal in patients, who exhibit well-controlled type 2 diabetes and coronary artery disease

**DOI:** 10.1186/s13098-019-0438-6

**Published:** 2019-05-31

**Authors:** Christian Anholm, Preman Kumarathurai, Anders Jürs, Lene Rørholm Pedersen, Olav Wendelboe Nielsen, Ole Peter Kristiansen, Mogens Fenger, Jens Juul Holst, Sten Madsbad, Ahmad Sajadieh, Steen Bendix Haugaard

**Affiliations:** 1 Department of Internal Medicine, Copenhagen University Hospital Glostrup, Nordre Ringvej 57, 2600 Glostrup, Denmark; 2 Department of Cardiology, Copenhagen University Hospital Bispebjerg, Copenhagen, Denmark; 3 Department of Clinical Biochemistry, Copenhagen University Hospital Hvidovre, Copenhagen, Denmark; 4 NovoNordisk Foundation Center for Metabolic Research and Department of Biomedical Sciences, Panum Institute, University of Copenhagen, Copenhagen, Denmark; 5 Department of Endocrinology, Copenhagen University Hospital Hvidovre, Copenhagen, Denmark; 6 Department of Endocrinology I, Copenhagen University Hospital Bispebjerg, Copenhagen, Denmark

**Keywords:** GLP1-receptor agonist, Diabetes mellitus type 2, Beta-cell function, Insulin sensitivity, Meal test, Insulin clearance, Glucagon

## Abstract

**Background:**

Hyperinsulinemia aggravates insulin resistance and cardio-vascular disease. How the insulinotropic glucagon-like peptide-1 receptor agonist liraglutide in a physiologic post-prandial setting may act on pancreatic alpha and beta-cell function in patients with coronary artery disease (CAD) and type 2 diabetes (T2DM) is less clear.

**Methods:**

Insulin resistant patients with established CAD and newly diagnosed well-controlled T2DM were recruited to a placebo-controlled, cross-over trial with two treatment periods of 12 weeks and a 2 weeks wash-out period before and in-between. Treatment was liraglutide or placebo titrated from 0.6 mg q.d. to 1.8 mg q.d. within 4 weeks and metformin titrated from 500 mg b.i.d to 1000 mg b.i.d. within 4 weeks. Before and after intervention in both 12 weeks periods insulin, C-peptide, glucose, and glucagon were measured during a meal test. Beta-cell function derived from the oral glucose tolerance setting was calculated as changes in insulin secretion per unit changes in glucose concentration (B_total_) and whole-body insulin resistance using ISI_composite_.

**Results:**

Liraglutide increased the disposition index [B_total_ × ISI_composite_, by 40% (n = 24, p < 0.001)] compared to placebo. Post-prandial insulin and glucose was reduced by metformin in combination with liraglutide and differed, but not significantly different from placebo, moreover, glucagon concentration was unaffected. Additionally, insulin clearance tended to increase during liraglutide therapy (n = 26, p = 0.06).

**Conclusions:**

The insulinotropic drug liraglutide may without increasing the insulin concentration reduce postprandial glucose but not glucagon excursions and improve beta-cell function in newly diagnosed and well-controlled T2DM.

*Trial registration* Clinicaltrials.gov ID: NCT01595789

## Background

The hyperglycemia in type 2 diabetes mellitus (T2DM) results from an imbalance between insulin secretion and insulin sensitivity [1] with impaired insulin action and an insufficient and delayed insulin response during meals as well as an inappropriate glucagon secretion [2]. Fasting as well as postprandial glucagon secretion increase progressively through the spectrum of impaired glucose tolerance to manifest T2DM [3]. The hyperglucagonemia is associated with hepatic insulin resistance [4] and an increased hepatic glucose production [5]. Elevated levels of non-esterified fatty acids (NEFA) resulting from adipose tissue insulin resistance may play a role in the development of peripheral as well as hepatic insulin resistance and may also impair beta-cell function in T2DM and in obese prediabetic individuals [6].

Metformin, which is recommended as first line therapy in patients with T2DM [7], has beneficial effects on HbA1c, body weight, cardiovascular mortality [7, 8] and insulin sensitivity [9]. However, metformin treatment has no effect on glucagon levels [10], whereas GLP-1 receptor agonists (GLP-1RA) have been suggested to inhibit glucagon secretion from alpha-cells [11, 12]. Furthermore, therapy with GLP1-RA is associated with a potentiation of glucose induced insulin secretion and a modest weight loss [13] and as a result it effectively reduces hyperglycemia in patients with T2DM [14]. This antihyperglycemic action of the GLP1-RA liraglutide is well-established in patients with longstanding not well-controlled diabetes [15–19]. The effect of GLP-1 receptor agonist on insulin sensitivity is still discussed [20–22], and its effect on insulin clearance is sparsely examined [23]. Newly diagnosed T2DM in patients with coronary artery disease (CAD) is associated with excess mortality [24]. Accordingly, it would be of interest how liraglutide may improve postprandial glycaemia and insulinemia in a population of newly diagnosed well-controlled T2DM subjects with CAD, particularly considering that liraglutide is insulinotropic.

The aims of the present study, therefore, were to evaluate effects of the GLP-1 RA liraglutide in combination with metformin on indices of alpha- and beta-cell function, insulin sensitivity and insulin clearance. This was evaluated using a mixed meal test in obese patients with newly diagnosed, well-controlled T2DM and high cardiovascular risk, a population in which efficacy of antidiabetic medication is essential and where several recent guidelines recommend GLP-1 RA, e.g. liraglutide as drug number 2 after metformin [7, 25].

## Methods

### Subjects

The inclusion criteria were stable coronary artery disease (CAD), body mass index (BMI) ≥ 25 kg/m^2^, age ≥ 18 and ≤ 85 years, and newly diagnosed (< 2 years) T2DM according to the criteria defined by the American Diabetes Association [26]. To be included, the patients were to be treated with diet, metformin or sulfonylurea alone or in combinations. All oral antidiabetic medications were stopped 2 weeks before baseline visit (Table 1). Exclusion criteria were, amongst others, previous treatment with a GLP-1RA or dipeptidyl peptidase-4 inhibitor. A comprehensive list of the exclusion criteria can be found elsewhere [27].

**Table 1:** Baseline characteristics of the study population, in median (IQR)

Variable	Units	Median	25th Pctl	75th Pctl	n
BMI	kg/m^2^	30.2	27.9	34.1	39
Age	years	64.0	58.0	68.0	39
Weight	kg	93.0	85.0	108.0	39
HbA1c^a^	mmol/mol	47 (6)			39
Fasting glucose	mmol/l	5.3	5.0	6.2	39
Fasting glucagon^a^	pmol/l	5.3 (3.5)			
Fasting C-peptide	pmol/l	1474	1006	1978	39
Fasting insulin	pmol/l	110	67	178	38
Fasting NEFA	mmol/l	0.392	0.298	0.455	39
AUC_glucose_	mmol/l × ^120^min	821.3	750.5	915.5	39
AUC_insulin_	pmol/l × ^120^min	33,173	25,350	45,625	38
AUC_ISR_	pmol/kg	969.5	811.1	1263.6	38
AUC_NEFA_	mmol/l × ^120^min	28.9	22.5	36.1	39
HOMA-IR		4.43	2.24	6.93	38
ISI_Composite_	l^2^/mg/microU	3.2	2.03	4.62	38
B-total		2.54	1.61	3.2	38
MCRi_kg_	l/kg/min	0.029	0.023	0.036	37
MCRi_total_	l/min	2.548	2.219	3.164	37
DI		8.06	5.71	12.91	37
IDR_basal_	pmol/kg/min	3.09	1.94	4.02	37
HEXi		0.30	0.22	0.36	37
Pre-study ADT^b^	n (%)				
Diet and lifestyle intervention	24 (62)				
Metformin	15 (38)				
Sulfonylurea	1 (3)				

^a^Mean (SD)

^b^Anti diabetic treatment

### Design

The study was an investigator-initiated, double-blind, randomized, placebo-controlled, cross-over trial. Details of the design, participants and intervention have been described previously [27].

Patients meeting the inclusion criteria were included consecutively and study drugs in subject boxes were sequentially numbered with a unique code and randomized by computer in a 1:1 randomization ratio by Novo Nordisk A/S and allocation sequence was concealed until all participants had completed the study [27]. Enrollment and assignment of participants were done at Department of Cardiology, Copenhagen University Hospital, Bispebjerg, Denmark. Patients were recruited from May 2012 to October 2014.

### Intervention

Liraglutide and metformin versus placebo and metformin in 12 plus 12 weeks with a 2-week wash-out period. The study period for each patient was 26 weeks and consisted of 4 major visits (at weeks 0, 12, 14 and 26); the wash-out was between weeks 12 and 14. Liraglutide dose was 1.8 mg once daily subcutaneously (titrated from 0.6 mg to 1.8 mg once daily within 4 weeks) and metformin 1 g twice daily orally (titrated from 500 mg twice daily to 1 g twice daily in 4 weeks) [27]. The last injection of liraglutide was in the morning on test days. Data collection was carried out in Department of Cardiology, Copenhagen University Hospital, Bispebjerg, Denmark.

### Endpoints

Beta-cell function (as measured by disposition index), insulin sensitivity, insulin clearance and responses of glucose, insulin, C-peptide, glucagon and NEFA during a 2-h mixed meal tolerance test, respectively. Treatment effects were evaluated by comparing results from the visits at initiation and end of each treatment period.

### Mixed meal test

A 375-g solid meal consisting of 60-g (46 E%) carbohydrates, 38-g (28 E%) protein and 16-g (26 E%) fat, equivalent to 550 kcal was consumed after an overnight fasting period of 10 h. Baseline blood samples were obtained at 10, 5 and 1 min before and 30, 60, 90 and 120 min after initiation of the test meal, which was to be finished within 15 min.

### Assay

Glucose measurements were carried out using an Accu-Chek Inform II meter (Roche, Swiss). Coefficient of variance (CV) was ≤ 3.3% for glucose levels > 4.2 mmol/l. Plasma insulin and C-peptide concentrations (pmol/l) were determined by the enzyme-linked immunosorbent assay (ELISA) (Siemens Healthcare Diagnostics, LA, California, USA), for insulin with an intra-assay CV of 3.3–5.5% and an inter-assay CV of 4.1–7.3% and for C-peptide with an intra-assay CV of 1.7–2.3% and an inter-assay CV of 2.9–4.8%. Plasma NEFA (mmol/l) was measured by an enzymatic test (Wako Chemicals, Neuss, Germany) with a median intra-assay CV of 1.5% and a median inter-assay CV of 7.5%. Glucagon concentrations in plasma were measured after extraction of plasma with 70% ethanol (vol./vol., final concentration). The antibody employed (code no. 4305) is directed against the C-terminus of the glucagon molecule and therefore mainly measures glucagon of pancreatic origin [28]. Standards were human glucagon and tracer was monoiodinated human glucagon (both gifts from Novo Nordisk, Bagsværd, Denmark). Sensitivity and detection limit is below 1 pmol/l, intra-assay CV below 6% at 20–30 pmol/l, and recovery of standard, added to plasma before extraction, about 100% when corrected for losses inherent in the plasma extraction procedure [29].

### Calculations

The composite measure of whole body insulin sensitivity (ISI_Composite_) [30] and the homeostasis model assessment of insulin resistance (HOMA-IR) [31] were determined as follows:

$$ISI_{composite} = \frac{10,000}{{\sqrt {\left( {FPG \times FPI} \right) \times \left( {\bar{G} \times \bar{I}} \right)} }}\quad HOMA{-}IR = \frac{{\left( {FPG \times FPI} \right)}}{405}$$

in which FPI is fasting plasma insulin (μU/ml), ${\bar{\text{I}}}$ is mean plasma insulin, FPG is fasting plasma glucose (mg/dl) and ${\bar{\text{G}}}$ is mean plasma glucose during meal test.

Prehepatic insulin secretion rates (ISR) (pmol/kg/min) were calculated from plasma C-peptide concentrations using the ISEC (Insulin SECretion) computer program [32]. This method is based upon the assumptions that C-peptide is not cleared by the liver and is co-secreted with insulin in equimolar amounts from the pancreas. The beta-cell response to changes in glucose during a meal test expresses the efficacy by which changes in plasma glucose concentrations stimulate insulin secretion. This relationship between changes in plasma glucose concentrations and ISR during the meal test was evaluated by cross-correlation analysis, and the slope of the regression lines (B_Total_) is a measure of the change in insulin secretion per unit change in glucose concentration, i.e. beta cell sensitivity to glucose or beta-cell responsiveness. Beta-cell function was defined as the product of beta-cell responsiveness and insulin sensitivity, i.e. the disposition index (D_i_) [33], i.e. assuming a hyperbolic association between beta-cell responsiveness and insulin sensitivity

$$D_{i} = B_{total} \times {\text{ISI}}_{composite}$$

We tested for the hyperbolic law in the basal state and found a R^2^ = 0.81 suggesting that the disposition index may be valid to use in the present study.

The trapezoidal rule [34] was used to calculate the area under the curve (AUC) for ISR, insulin, glucose and NEFA concentrations. The integer of ISR (AUC − ISR_0–120 min_) represents the total amount of insulin secreted throughout the meal test while the integer of insulin concentration profiles during the meal test (AUC − insulin_0–120 min_) represents both insulin secretion and insulin clearance. Thus, the ratio of these integers adjusted for total body mass reflects insulin clearance (MCRi):

$$MCRi = \frac{{AUC - ISR_{0 - 120\,min} }}{{AUC - Insulin_{0 - 120\,min} }} \times Total\,body\,mass$$

Plasma insulin levels are at steady-state in the basal period, therefore the posthepatic insulin delivery rate (IDR_Basal_) can be calculated as [35]:

$$IDR_{Basal} = insulin_{Basal} \times MCRi$$

The ratio of the IDR_Basal_ to the ISR_Basal_ is the fraction of insulin not extracted by the liver, therefore the hepatic extraction of insulin (HEXi) is calculated as [35]:

$$HEXi = 1 - \frac{{IDR_{Basal} }}{{ISR_{Basal} }}$$

To evaluate the efficacy of insulin to suppress NEFA production [36] we divided ∆AUC − NEFA_0–120 min_ by ∆AUC − insulin_0–120 min_, (denoted NEFA_ins_).

### Statistical analysis

Data are reported as median (IQR) or mean (SD). Students’ paired t-test was used for normally distributed data when comparing groups. In non-normally distributed data, Wilcoxon’s Signed Rank test was used. A two-sided value of p < 0.05 was considered statistically significant. Comprehensive details on the power calculation are published elsewhere [27]. Statistical analyses were performed with SAS 9.4 (SAS Institute Inc., Cary, North Carolina, USA). The study was approved by the Regional Committee on Biomedical Research Ethics of the Capital Region of Denmark and was carried out in accordance with the International Conference on Harmonization—Good Clinical Practice standards and informed consent was obtained from all participants. The study protocol was registered at Clinicaltrials.gov with ID: NCT01595789.

## Results

### Participants

**Fig. 1:**
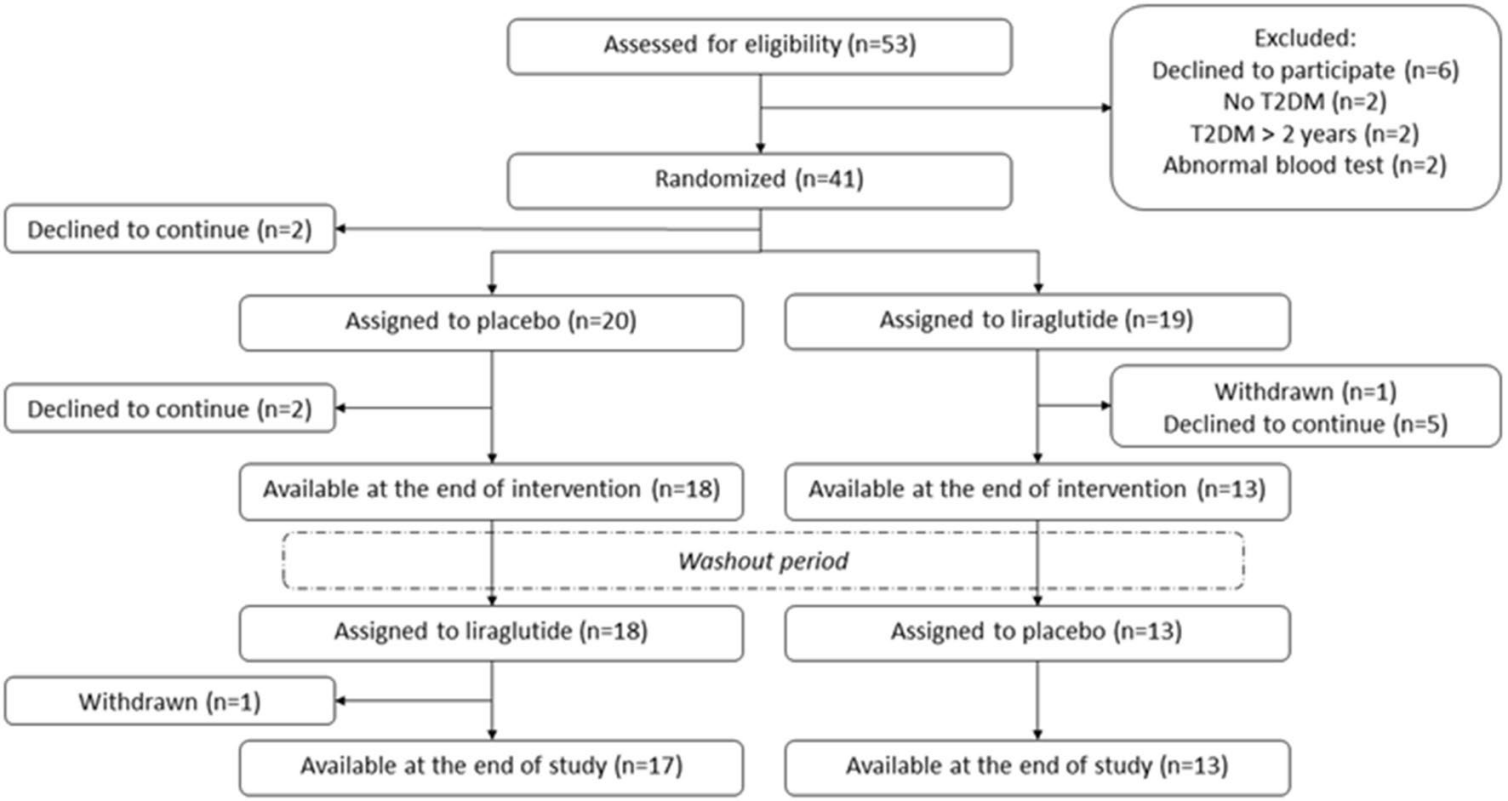
Screening, randomization and follow-up [39]

Of the 41 patients randomized, two patients declined to participate before first visit and nine patients discontinued the study (Fig. 1). Twenty-eight patients completed all study visits. Two patients treated with placebo in the first period could not attend visit 3 therefore data from visit 2 were carried forward and used as baseline for the second period. Thus, thirty participants were included in the paired analyses. Eliminating data from the two patients did not influence the main results. Baseline characteristics are found in Table 1. Calculations of indices were limited by missing values in a few patients, thereby leading to patients < 30 (Tables 2, 3). At baseline mean HbA1c was 47 [6] mmol/mol.

**Table 2:** Indices of beta-cell function, insulin sensitivity and clearance

Variable (unit)	Baseline	n	Placebo	n	p	Liraglutide	n	p	Difference	n	p
B_Total_	2.72 (2.00; 3.24)	29	− 0.14 (− 0.59; 0.37)	28	0.16	0.2 (− 0.54; 0.79)	25	0.42	0.25 (− 0.35; 0.93)	24	0.24
DI	8.23 (5.87; 12.93)	28	1.77 (− 1.83; 4.91)	27	0.06	6.0 (2.78; 9.23)	25	< .0001	3.35 (− 0.51; 9.29)	24	0.0005
IDR_basal_ (pmol/kg/min)	3.2 (2.18; 4.37)	29	− 0.28 (− 0.92; 0.25)	28	0.1	− 0.16 (− 1.13; 0.27)	26	0.22	0.13 (− 1.01; 0.84)	25	0.84
ISI_Composite_ (L^2^/mg/microU)	3.15 (2.03; 4.5)	30	0.73 (− 0.05; 2.31)	29	0.0005	1.68 (0.57; 3.34)	27	< .0001	0.26 (− 0.81; 2.26)	27	0.14
HOMA-IR	4.43 (2.75; 6.93)	30	− 0.74 (− 2.06; − 0.12)	28	0.007	− 1.32 (− 2.88; − 0.25)	26	0.0003	− 0.39 (− 1.98; 0.79)	26	0.43
MCRi_kg_ (ml/kg/min)	29.35 (23.47; 35.07)	29	1.65 (− 0.99; 4.38)	28	0.08	3.56 (0.29; 7.02)	27	0.005	2.92 (− 2.47; 6.55)	26	0.06
MCRi_total_ (L/min)	2.54 (2.17; 3.33)	29	0.11 (− 0.10; 0.36)	28	0.15	0.27 (0.02; 0.37)	26	0.02	0.25 (− 0.24; 0.49)	25	0.13
HEXi	0.293 (0.208; 0.359)	29	0.024 (− 0.089; 0.083)	28	0.69	0.017 (− 0.073; 0.106)	26	0.72	0.058 (− 0.137; 0.146)	25	0.97

Units are mmol/l × ^120^min except for insulin secretion rate (ISR) measured i pmol/kg. Data reported as median (IQR)

**Table 3:** Area under the curve (AUC) in median (IQR), units are mmol/l × ^120^min except ISR measured in pmol/kg

Variable	Baseline	n	Placebo	n	p	Liraglutide	n	p	Difference	n	p
Glucose	804.3 (735.5; 911.3)	31	− 75.9 (− 143.5; − 29.0)	29	0.0003	− 105.6 (− 213.0; − 40.8)	28	< .0001	− 44.8 (− 122.3; 33.8)	27	0.09
Insulin	36,103 (25,435; 48,380)	30	− 8530 (− 14,630; 1915)	29	0.006	− 11,270 (− 19,382; − 1145)	27	0.002	− 2195 (− 7745; 4250)	27	0.45
ISR	999 (840; 1281)	30	− 205 (− 360; − 32)	29	0.001	− 102 (− 260; 105)	26	0.08	69 (− 25; 247)	25	0.11
NEFA	29.1 (22.6; 37.9)	31	− 2.8 (− 7.5; 2.9)	30	0.06	2.7 (− 5.5; 13.4)	27	0.26	6.5 (− 5.7; 16.9)	27	0.13

### Alpha-cell function

**Fig. 2:**
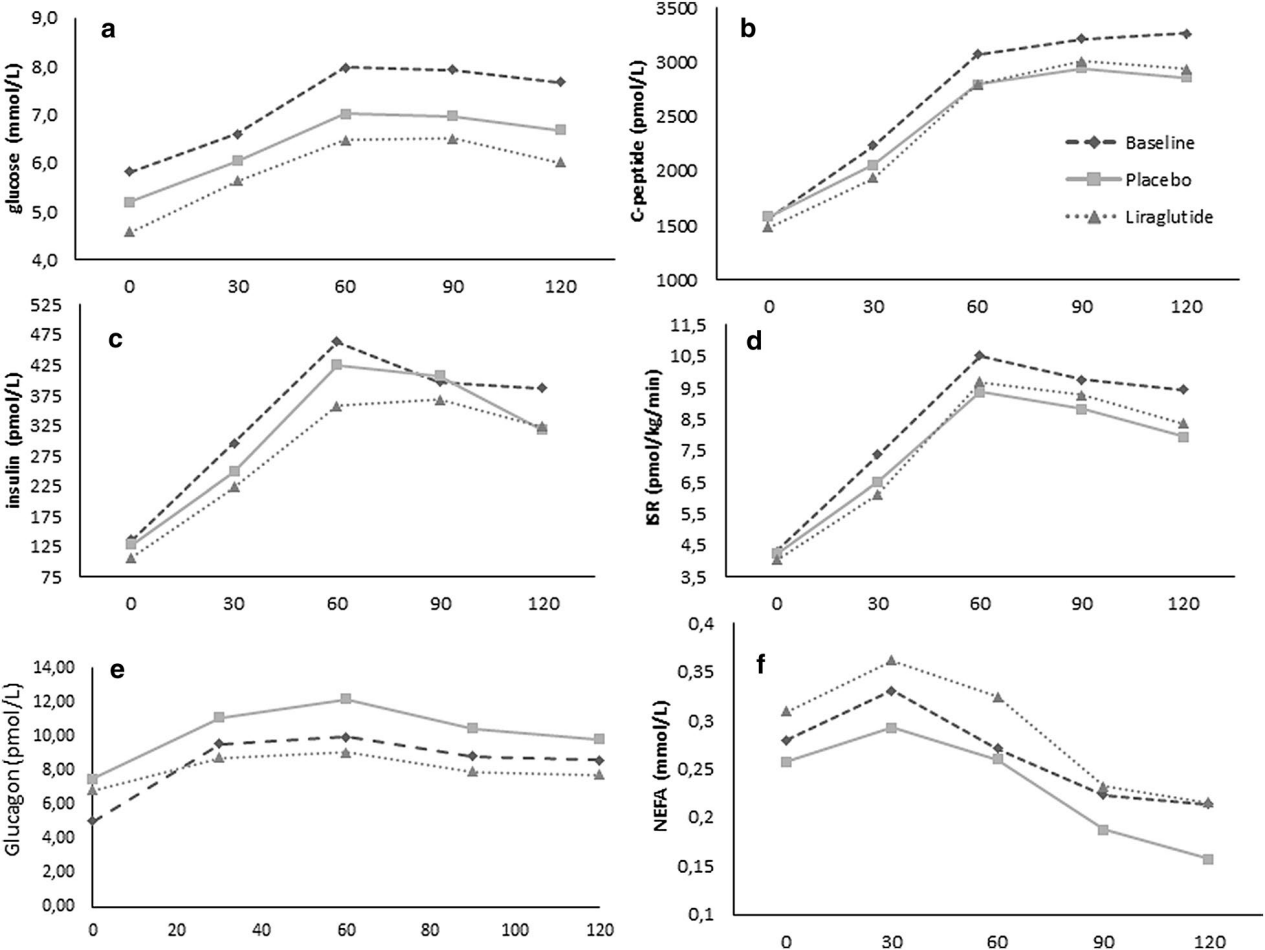
Plasma levels of glucose (**a**), C-peptide (**b**), insulin (**c**), insulin secretion rates (**d**), glucagon (**e**) and NEFA (**f**) during meal test

Baseline fasting p-glucagon was 5.3 (3.5) pmol/l. Both placebo and liraglutide treatment increased fasting glucagon levels to 7.5 (3.8) pmol/l (p < 0.001) and 6.8 (3.1) pmol/l (p < 0.002), respectively, but with no significant difference between periods (p < 0.7). AUC_glucagon_ was not significantly changed by placebo treatment [1138 (663) to 1283 (637) pmol/l × ^120^min (p = 0.07)], and neither did liraglutide treatment affect AUC_glucagon_ [992 (516) to 995 (417) pmol/l × ^120^min (p = 0.64)], and no difference between treatments was observed (p < 0.4). AUC_insulin/glucagon_ was reduced by placebo (p = 0.01) but not by liraglutide treatment (p < 0.06) and with no difference between treatment periods (p = 0.09) (Fig. 2).

### Insulin sensitivity

ISI-composite (ISI_comp_) was increased in both treatment periods: placebo; 0.73 (− 0.05 to 2.31) l^2^/mg/microU (p < 0.0005) and liraglutide; 1.68 (0.57 to 3.34) l^2^/mg/microU (p < 0.0001), with no significant differences between treatments (p = 0.14). HOMA-IR was reduced significantly in both placebo and the liraglutide period but with no differences between treatments (Table 2).

### Beta-cell function and plasma glucose

AUC_glucose_ was reduced in both placebo and liraglutide periods by − 76 (− 144 to − 29) mmol/l × ^120^min and − 106 (− 213 to − 41) mmol/l × ^120^min, p = 0.0003 vs. p < 0.0001, respectively, with no difference between treatments (Table 3 and Fig. 2). Insulin and C-peptide responses are depicted in Fig. 2. Analysis of AUC’s showed significant reductions of AUC_insulin_ in both placebo and liraglutide periods with no significant difference between treatments (p < 0.46) (Table 3). Additionally, the AUC insulin/glucose ratio was not increased by liraglutide treatment: − 50 (312) pmol/mmol (p = 0.4).

**Fig. 3:**
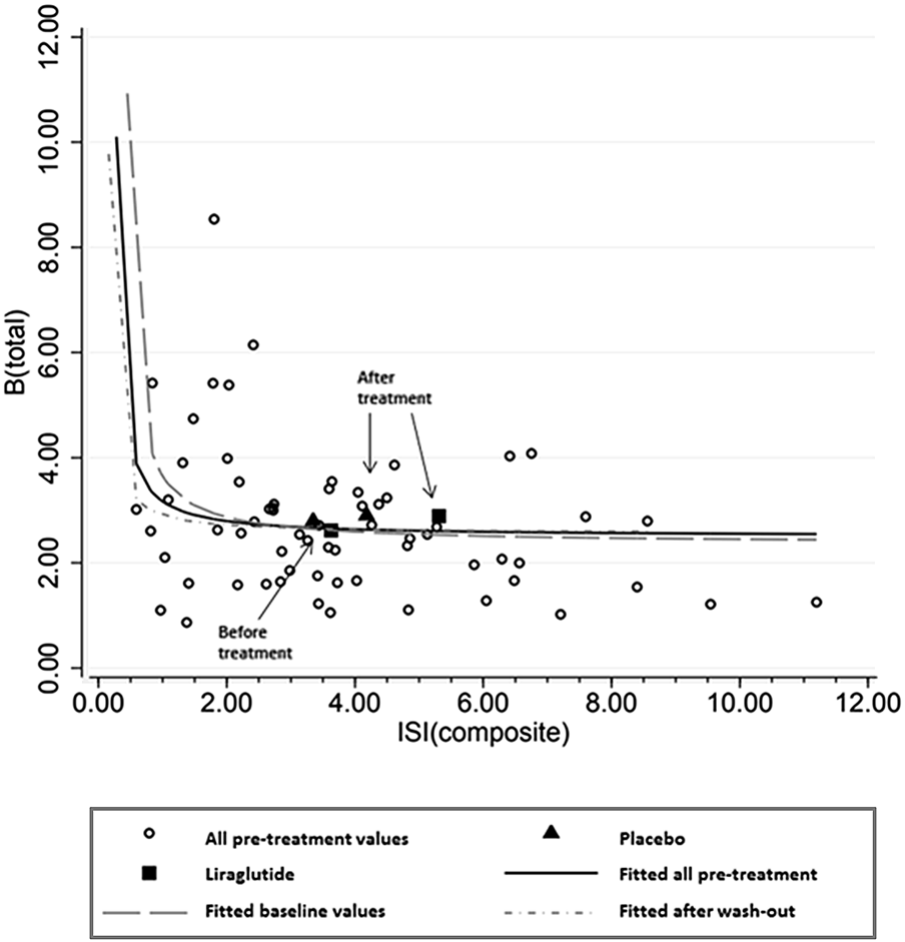
The hyperbolic relationship between B_total_ and ISI_Composite_—Open circles represents all pretreatment values, i.e. baseline values combined with values after wash-out before beginning of period 2. The hyperbolic function is evident for both groups and for all pretreatment values. Fitted (all); R^2^ = 0.81, fitted (baseline):R^2^ = 0.8, fitted (washout); R^2^ = 0.83. Treatment effect is indicated by square and triangle (liraglutide vs. placebo), beginning and end of treatment are depicted by arrows

Basal insulin delivery rates (IDR_basal_) were not affected by either treatment (Table 2). AUC_ISR_ was significantly reduced only in the placebo period, with no additional change observed with liraglutide treatment (Table 3). Paired analysis (n = 25) did not reveal significant differences in ISR between placebo and liraglutide treatment (Table 3). Beta-cell responsiveness (B_total_) did not differ between treatments (Table 2). Disposition index (DI) was improved in the liraglutide period; 6.0 (2.78 to 9.23), p < 0.0001, whereas placebo treatment did not affect DI significantly. Paired analysis revealed an effect of liraglutide therapy of 3.35 (− 0.51 to 9.29), p = 0.0005, improving baseline value by 40% (Table 2) towards a less diabetic state (Fig. 3). Of importance we observed a strong baseline hyperbolic association between ISI_comp_ and B_total_ of R^2^ = 0.81, which comply with the data that was obtained in the original method study on this relationship using intravenous glucose defining the beta-cell function [37].

### Insulin clearance

Baseline fasting insulin was inversely correlated to MCRi_kg_ (R^2^ = 0.58, p < 0.0001), MCRi_total_ (R^2^ = 0.33, p = 0.001) and HEXi (R^2^ = 0.31, p < 0.002). In the placebo period MCRi_kg_ was numerically but non-significantly increased but the combination of liraglutide and metformin increased MCRi_kg_ significantly by 3.56 (0.29 to 7.02) × 10^−3^ L/kg/min (p = 0.005) with a borderline difference between groups (p = 0.06) (Table 2). Improvement in ISI_comp_ was associated with reduction in MCRi_kg_ (R^2^ = 0.21, p = 0.02) and MCRi_total_ (R^2^ = 0.24, p = 0.01). There were no associations with HOMA-IR. Baseline HEXi was reduced as compared to normoglycemic subjects who usually clear app. 50% or more during the first pass of the liver [38]. These patients exhibit a HEXi of only 29% (21 to 36%) and liraglutide therapy had no effect on HEXi.

A subgroup analysis of the 24 patients with complete data set with respect to: B_total_, ISI_comp_, DI, HOMA-IR, MCRi_kg/total_ and HEXi confirmed the results outlined previously. However, regarding MCRi_kg_ the effects of liraglutide and placebo were comparable to the analysis based on all patients, nevertheless, the placebo corrected difference reached statistical significance (p = 0.03).

### NEFA

Baseline fasting NEFA did not correlate to weight, BMI or HOMA-IR. AUC_NEFA_ did not change significantly in the placebo period in contrast to the liraglutide period in which AUC_NEFA_ increased significantly, but the difference between the groups was not statistical different (p = 0.13) (Table 3). The amount of insulin required to suppress NEFA production (NEFA_ins_) was not changed significantly in placebo period but was reduced in liraglutide period; − 470 (− 1790 to 10) nmol/pmol, p < 0.05 (Fig. 2).

### Hba1c and body weight

HbA1c at 47 [6] mmol/mol was reduced by − 3.3 (6.51) mmol/mol, p < 0.01 and body weight at 93 (85 to 108) kg was reduced by − 2.7 (− 6.7 to − 0.6) kg, p = 0.0004, both corrected for placebo treatment.

### Explanatory variables and carry-over effect

We found no correlations between weight loss and ISI_comp_; placebo (R^2^ = 0.01; p > 0.7) and liraglutide (R^2^ = 0.06; p > 0.2) or weight loss and DI; placebo (R^2^ < 0.01; p > 0.7) and liraglutide (R^2^ = 0.08; p > 0.2). The variance of weight loss was not associated with baseline weight, BMI, age, sequence of treatment, and differences in treatment duration [39]. The presence of a possible carry-over effect was estimated using sum values by the t-test [40], but we did not find any significant carry-over effect between periods with respect to weight loss (p = 0.4) [39], DI (p = 0.4), B_total_ (p = 0.1) or ISI_comp_ (p = 0.1).

### Post-hoc power calculation

Power calculation was based on improvement of disposition index during i.v. glucose test as reported earlier [39]. In the present paper we report secondary outcomes, and post-hos power analysis were done. Given a power of 80% and a level of significance of p < 0.05 we would be able to detect an increase in Disposition index on > 40% with n = 34 patients in paired analysis. B_Total_ an increase on 20% with 28 patients, however, regarding ISI_Composite_ we would need n = 134 patient for detecting the same change in paired analysis.

### Compliance and safety

Use of the study drugs (metformin, liraglutide and placebo) was counted, and compliance to liraglutide/placebo and metformin was > 90% of prescribed dosages with no significant differences between treatment periods. Adverse event frequency was higher in the active treatment periods predominantly due to gastrointestinal side effects. Serious adverse events were observed in a total of 9 cases; 3 in the active period, 4 in the placebo period and 2 in the wash-out period. A detailed description of adverse events is published elsewhere [41].

## Discussion

This meal test study of newly diagnosed well-controlled patients with T2DM and established CAD showed that liraglutide combined with metformin versus metformin (+placebo) significantly improved beta-cell function with a trend towards improved insulin sensitivity. Of note, the combination of the insulinotropic liraglutide and metformin reduced post-prandial insulin levels in face of a reduced glucose excursion, which is a new finding in this setting.

In this population we have already shown by use of an non-physiologic intravenous glucose tolerance test a significant improvement in hyperglycemia and a significant increase in insulin levels [39], which was expected as liraglutide is an insulin secretagogue [12].

However, in the present study we report data from a mixed meal test where we observed a reduction of insulin as well as glucose levels during this physiological test. The effect of liraglutide on beta-cell responsiveness (B_total_) was neutral. These finding might in part be explained by the very well-controlled hyperglycemia with a mean HbA1c of 47 mmol/mol and a baseline fasting plasma glucose on 5.3 mmol/l. Despite these findings the improvement in disposition index is substantial in the present study, just as seen in the non-physiological setting using intravenous glucose as beta-cell stimulation in combination with the so called “minimal model” in the same patients [39]. The novelty of the present physiologic study is that despite reduction of insulin levels, liraglutide (combined to metformin) retains its strong effect on beta-cell function (i.e. disposition index) in patients with well-controlled T2D and established CAD.

Furthermore, the combination therapy improved insulin clearance and insulin sensitivity, both hepatic and peripheral. In our setup we evaluated basal level of hepatic insulin extraction but did not find effect of liraglutide on this, however basal level was reduced compared to which was previously reported on glucose intolerant subjects [42]. Reduced hepatic extraction is the primary cause of high levels of circulating insulin after a glucose load [42] and in normoglycemic subjects hepatic extraction is suppressed up to 30% for ≤ 150 min following a glucose load [38] and 40–50% of secreted insulin is extracted during a standard meal [38]. A body weight loss of > 10 kg increased hepatic extraction of insulin [43] and the weight loss in our study was only < 3 kg.

A recent Japanese study used a mixed meal test and compared metformin and liraglutide as monotherapy in patients with T2DM, and was able to demonstrate improvements in beta-cell function (measured as disposition index) by liraglutide compared to no effect following metformin therapy [44]. An earlier acute study of 11 subjects with type 2 diabetes and with a HbA1c of 6.5 ± 0.6% a single dose of liraglutide revealed an increase in fasting ISR but post meal AUC_ISR_ was unaltered in contrast to AUC_glucose_ which was markedly reduced, indicating an improved beta-cell function [45].

The effect on the alpha-cell function was unexpected; fasting glucagon levels increased during liraglutide and placebo treatments but neither treatment led to lower glucagon responses during meal test.

Data from the *liraglutide effect and action in diabetes* (LEAD) studies are conflicting with respect to the effect on fasting glucagon levels; in LEAD-3 a decrease of fasting glucagon was found but no effect was observed in LEAD-4 [15, 17]. The absent suppression of glucagon in the present study may indicate a somewhat different action of liraglutide on alpha-cell function compared to native GLP-1, which may relate to the duration of treatment, since a short term period of GLP-1-infusion inhibits glucagon secretion [46], whereas a long term infusion does not retain the same glucagon suppressive effect [20]. However, a previous study on patients with early stage T2DM revealed somewhat ambiguous results [47, 48], and it is suggested that attention must be paid to the performance of the assay used to measure glucagon, in order to obtain valid results [29]. Additionally, the observed differences in glucagon response can to some extend be caused by differences in the composition of the test meals [49] and whether a the oral challenge is a mixed meal or glucose [50]. It is indicated that postprandial glucagon levels are increased after at mixed meal as compared to glucose alone [50]. We therefore speculate that the present setting may implicate a somewhat different glucagon response compared to an oral glucose challenge or, alternatively that subjects with more advanced disease might reveal a different picture.

In T2DM the suppression of NEFA by insulin is diminished and increased NEFA levels impair insulin action and insulin secretion [1]. In the present study liraglutide did not change suppression of NEFA compared with metformin but less insulin was needed to suppress postprandial NEFA in the liraglutide arm.

The present results suggest that in well-controlled subjects with type 2 diabetes the positive effect of liraglutide treatment on beta-cell function is clinically relevant but the effect on alpha cell function is subtle. Additionally, the combination of liraglutide with metformin improves insulin sensitivity and clearance and no effect on post-meal hepatic extraction of insulin. However, it is emphasized that liraglutide may produce another metabolic response in patients with less well-controlled and more advanced diabetes patients, which limits the generalization of this study.

A limitation of the study was the relatively few samples and a short duration of the meal test, since longer protocols of longer duration including a higher number of samples will reveal a more detailed picture of early as well as late insulin secretion and alpha-cell function as well as NEFA metabolism in response to a meal test [51]. The disposition index calculated from the mixed meal test remains to be validated. The assumption is that the beta-cell adaption to ambient insulin resistance follows a hyperbolic law (y = 1/x), and that the product AIR glucose × Si (the disposition index), therefore, is constant in people with normal beta-cell function. The disposition index in subjects with type 2 diabetes is used to obtain a correct estimate of the beta-cell function it relation to the prevailing degree of insulin resistance of the individual. In the present study we tested for the hyperbolic law in the basal state and found a R^2^ = 0.81 suggesting that the disposition index may be valid. However, post hoc power analysis indicates that changes in some of the indices reported, especially regarding ISI_Composite_ could flawed due to the study being underpowered. Nevertheless, this explorative study could indicate a more metabolic flexibility of liraglutide treatment than previously acknowledged. The data presented her warrants further experiments in a greater population.

The data presented here were obtained during a relatively short-term treatment period and demonstrated the effects of liraglutide in newly diagnosed patients. Data from real-world clinical practice indicates that the treatments effects we observed are similar to what was observed in clinical trials [52]. The present study aimed to reveal treatment effects on pathophysiological features of early T2DM and CAD, yet we speculate that effects in more advanced disease might reveal a different picture, which might add to our knowledge behind the long term positive cardiovascular profile of liraglutide [53]. In accordance with clinical trails the side-effect profile of liraglutide was relatively low and comprised mainly of gastrointestinal events, which often resolves within 4 weeks of therapy [41, 54]. The present study did not indicate pathophysiological pathways, which could indicate possible adverse long-term effects. Adverse out-come in our trial does not differ substantially from clinical trials [41, 54]. Additionally, recent data indicates that liraglutide treatment is not associated with an increased risk for pancreatitis or pancreatic cancer [55].

## Conclusions

In patients with well-controlled T2DM and CAD liraglutide treatment significantly improved disposition index as a measure of beta-cell function but has no effect on alpha-cell function. Combined with metformin, liraglutide reduced ambient insulin levels and improved insulin sensitivity and insulin clearance in a physiologic meal test setting suggesting that liraglutide is as a flexible insulinotropic drug in these patients.

## Data Availability

The datasets used and/or analysed during the current study are available from the corresponding author on reasonable request.

## Abbreviations

T2DM: type 2 diabetes mellitus
NEFA: non-esterified fatty acids
GLP1-RA: glucagon-like peptide-1 receptor agonist
CAD: coronary artery disease
BMI: body mass index
CV: coefficient of variance
HOMA-IR: homeostasis model assessment of insulin resistance
ISR: insulin secretion rates
ISEC: Insulin SECretion (computer program)
DI: disposition index
AUC: area under the curve
MCRi: insulin clearance
IDR: insulin delivery rate
HEXi: hepatic extraction of insulin
IQR: interquartile range
SD: standard deviation
LEAD: liraglutide effect and action in diabetes
